# The enhanced photo absorption and carrier transportation of InGaN/GaN Quantum Wells for photodiode detector applications

**DOI:** 10.1038/srep43357

**Published:** 2017-02-27

**Authors:** Haojun Yang, Ziguang Ma, Yang Jiang, Haiyan Wu, Peng Zuo, Bin Zhao, Haiqiang Jia, Hong Chen

**Affiliations:** 1 Key Laboratory for Renewable Energy, Beijing Key Laboratory for New Energy Materials and Devices, Beijing National Laboratory for Condensed Matter Physics, Institute of Physics, Chinese Academy of Sciences, Beijing 100190, China

## Abstract

We have conducted a series of measurements of resonantly excited photoluminescence, photocurrent and photovoltage on InGaN/GaN quantum wells with and without a p-n junction under reverse bias condition. The results indicate that most of the resonantly excited photo-generated carriers are extracted from the quantum wells when a p-n junction exists, and the photon absorption of quantum wells is enhanced by the p-n junction. Additionally, the carrier extraction becomes more distinct under a reverse bias. Our finding brings better understanding of the physical characteristics of quantum wells with p-n junction, which also suggests that the quantum well is suitable for photodiode detectors applications when a p-n junction is used.

The light-to-electricity conversion process of III-nitride materials and devices is the essential foundation for visible and ultraviolet photodetectors that are potentially applied in light communication, medical care and smoke alarm^1^,^2^,^3^,^4^. However, the conversion efficiency is still low due to difficulties for obtaining low density of dislocations and high conductive of epitaxial films^1^,^5^,^6^. Generally, the conversion efficiency is composed of the photo absorption and carrier extraction efficiency. The p-n junction is one of the most important structures for carrier extraction, of which the feature characteristic is collecting the electrons and holes from depletion region. When a p-n junction is under illumination, the photo-excited electrons and holes are drifted to the opposite sides of the p-n junction by the built-in field, thus an electrical response generates for photodiode detectors^7^,^8^. According to this, the most popular photodetectors are based on p-i-n structures.

Extending the absorption region (i-layer) width is a general method of enhancing the absorption efficiency for a photodetector device. However, a large lattice mismatch between InN, GaN and AlN becomes an impediment to the growth of high-quality thick hetero-epitaxial layer due to increased biaxial strain in wurtzite structures^9^. It brings high-density of threading dislocations that can seriously deteriorate the device performance^10^. Comparing with bulk materials, multiple quantum wells (MQW) structure can modulate the interface strain thus improves the crystalline quality.

According to established theories, the photo-generated carriers caused by interband absorption of quantum wells will relax to ground state and cannot form the photocurrent owing to the confinement. Hence, to date, most of the III-nitride UV photodetectors have relied on bulk-like epilayers. Recently, some results have been presented about photodetectors made using of quantum wells by interband absorption^11^,^12^,^13^,^14^,^15^,^16^. It was thought that MQW-based detectors have different advantages over bulk devices, such as flexibility to tune the detection edge^12^,^15^. Even so, there is yet a big lack in the investigation of multiple-quantum-well photodetectors. Here, we find that when the p-n junction is applied under short circuit, most of the resonantly excited carriers can escape from the wells and form photocurrent rather than relax to ground state for recombination. If a reverse bias is applied on the quantum wells with p-n junction, this carrier extraction effect is enhanced, which may be a new mechanism to use quantum wells for photodetectors applications.

Two samples with 10-period InGaN/GaN multiple quantum wells grown on c-plane sapphire substrates by Metal Organic Vapor Phase Epitaxy (MOVPE) are prepared. The multiple quantum wells covered by p-GaN is named sample A (with p-n junction) and the one covered by n-GaN is named sample B (without p-n junction), as showed in Fig. 1. A laser with a wave-length of 405 nm, i.e. the photon energy of 3.06 eV is used to resonantly excite the carriers in multiple quantum wells-due to the photon energy is between the band gap of GaN barriers and InGaN wells^17^,^18^.

## Results

**Figure 2:**
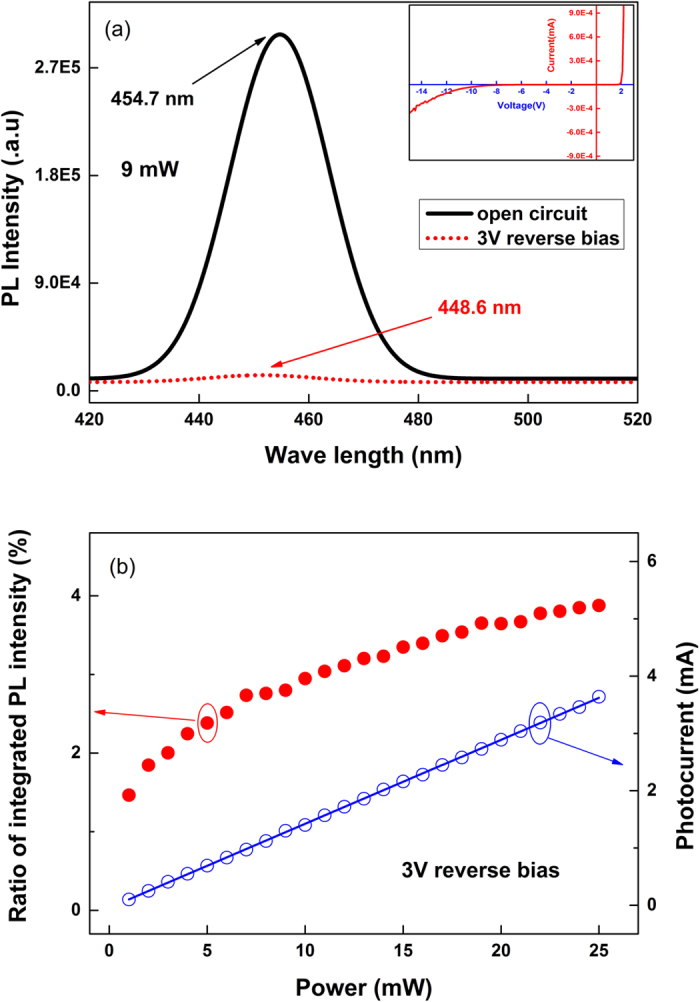
The photo-electric characteristic of sample A (with p-n junction). (**a**) PL spectra of sample A obtained with an excitation power of 9 mW in open circuit voltage conditions and under a reverse bias of 3 V. The insert reports the current-voltage characteristics of sample A. (**b**) The dependence of the (3 V) reverse-bias photocurrent and the ratio of integrated PL intensities (PL intensity under reverse bias divided by PL intensity in open circuit voltage conditions) on the excitation power.

PL measurement of sample A under open circuit and 3 V reverse bias condition are taken and shown in Fig. 2(a). The PL intensity under 3 V reverse bias condition decreases dramatically and only accounts for 2.8 percent of that under open circuit for an excitation power of 9 mW. The peak wavelength of PL shifts from 454.7 nm under open circuit to 448.6 nm under 3 V reverse bias condition. At the same time, an open circuit voltage of 2.42 V and a photocurrent of 1.46 mA under 3 V reverse bias condition are obtained. According to the current-voltage characteristics shown in insert of Fig. 2(a), sample A demonstrates good rectifying properties with a low circuit current under reverse bias. Hence, it indicates that the photocurrent is generated from the multiple quantum wells in the p-n junction because most of the photo-generated carriers are extracted from wells rather than relax to ground state for recombination when illumination is applied. Figure 2(b) shows the power-dependent photocurrent and ratio of integrated PL intensity of sample A under 3 V reverse bias. The linear dependent of photocurrent with the incident power further confirms that the photocurrent comes from the quantum wells. The ratio of integrated PL intensity under 3 V reverse bias condition to that under open circuit changes from 1.46% to 3.88% with the incident power, which means that only minority of the photo-carriers are confined in quantum wells for recombination under 3 V reverse bias condition. The above photo-electric characteristics of the quantum wells with a p-n junction show that even though the incident photon energy is lower than the barrier band gap, most of the photo-generated carriers escape from wells rather than relax to ground confined state for recombination when the p-n junction is under reverse bias. When sample A was under open circuit, firstly the photo-generated electrons and holes were extracted to the opposite sides of the p-n junction, thus the open voltage generated. The open voltage resulted in an electric field, which have an opposite direction with the built-in field. The electric field resulted from open voltage would counteract the built-in field and a new equilibrium established instantaneously, then most of photo-generated carriers stopped escaping from the quantum wells but were confined for radiative recombination. So the sample A under open circuit showed a large PL peak and an open circuit voltage.

**Figure 3:**
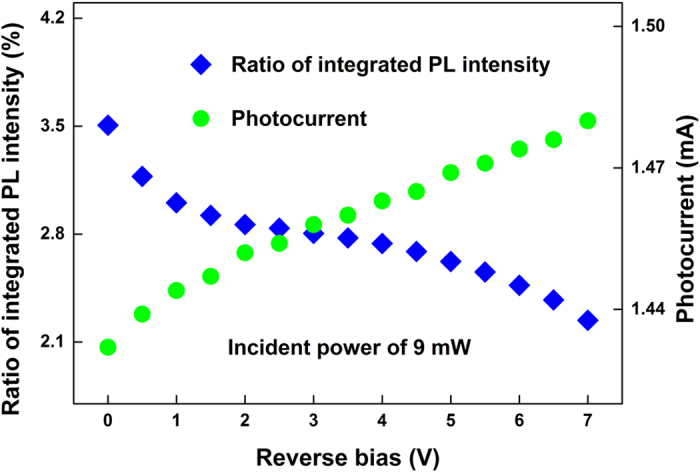
Photocurrent and ratio of the integrated PL intensity for sample A under reverse bias.

Furthermore, the photo-electric characteristics of sample A under varied reverse bias are studied in details. As Fig. 3 shows, the photocurrent increases from 1.42 mA to 1.48 mA when the reverse bias changes from 0 V to 7 V with an excitation power of 9 mW, the corresponding ratio of integrated PL intensity under reverse bias condition to that under open circuit decreases from 3.5% to 2.2%. It is worth noting that a photocurrent of 1.42 mA generates even under 0 V bias, which means most of the excited carriers escape from the quantum wells when the p-n junction is under short circuit. The increasing of photocurrent and decreasing of integrated PL intensity with reverse bias shows that an extra reverse bias enhances the carriers escaping effect further, since it enhances the electric field in the p-n junction.

**Figure 4:**
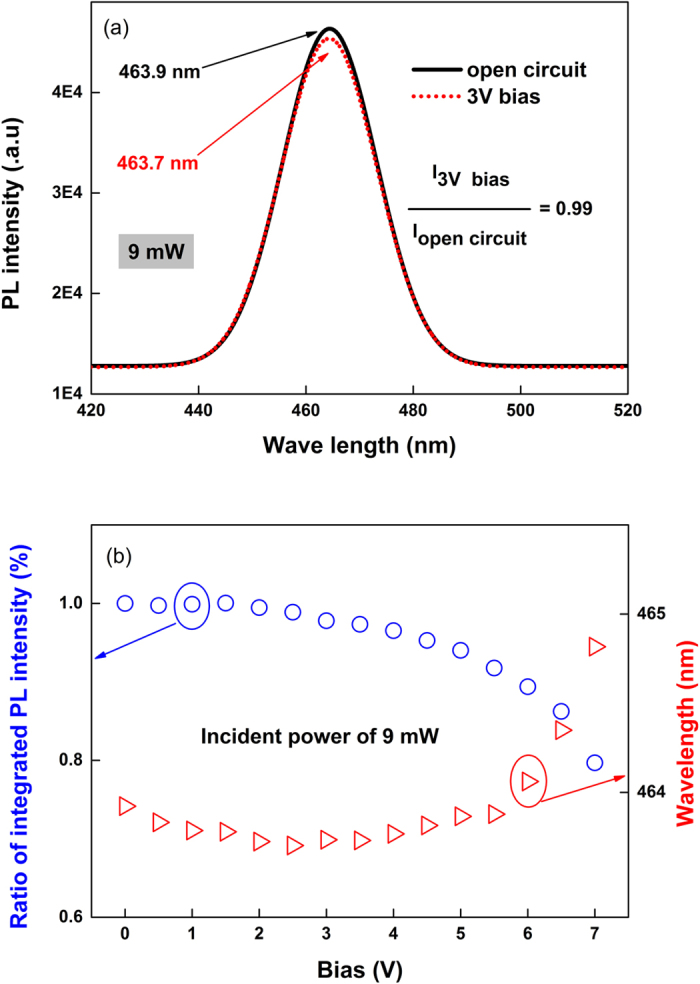
The photo-electric characteristics of sample B (without p-n junction). (**a**) Comparison between the PL spectra obtained with an excitation power of 9 mW in open circuit voltage conditions and under a bias of 3 V. (**b**) the bias-dependent peak position in the PL spectrum and for the ratio of integrated PL intensities.

The same comparison, with and without an external bias, is applied to sample B (a quantum well structure without p-n junction) to study the role of p-n junction in our experiment. For an excitation power of 9 mW, the integrated PL intensity under 3 V bias condition decrease by 1% compared with that under open circuit, and the peak positions are nearly identical (463.9 nm and 463.7 nm) as shown in Fig. 4(a). The InGaN quantum well layer in our samples suffered a piezoelectric polarization field pointed to the direction of sapphire substrate. When sample B is under bias condition, the external field is opposite with the polarization field of quantum wells. Hence, the external field should counteract the polarization field first and the PL peak showed a little blue-shift accordingly shown as Fig. 4(b). The ratio of integrated PL intensity under bias condition to that under open circuit changes slightly with the bias, which only decreases to 78% when the bias is up to 7 V. This means only 22% of the photocarriers lost (comparing with photoluminescence under open circuit), much less than that of the p-n junction under short circuit and reverse bias. This carriers loss can be partially attributed to the decreasing of recombination caused by the quantum confined Stark effect, where the external filed slant the energy band with the increasing of external bias^19^. It is confirmed by the red shift of PL peak position from 463.9 nm under open circuit to 464.8 nm under 7 V bias.

## Discussion

The quantum efficiency defines as the number of electron hole pairs extracted per incident photon. It is equal to the current responsivity times the photon energy of the incident radiation^15^:


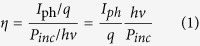


where *P*_inc_ is the incident optical power in watts, *I*_ph_ is the photocurrent, h is the Plank constant and ν is the radiation frequency. Sample A showed a photocurrent of 1.46 mA under 3 V reverse bias with an incident power of 9 mW, thus the quantum efficiency is calculated to be 49.6% accordingly. Considered the total thickness about 25 nm of quantum wells, an absorption coefficient of 2.7 × 10^5^ cm^−1^ is obtained, which is larger than the usually reported of 1 × 10^5^ cm^−1^ for InGaN film^20^,^21^. Hence the photo absorption of quantum wells was enhanced by the p-n junction. Taking a more comparison of sample A with sample B, it can be concluded that the p-n junction makes most of the resonant excited photo-generated carriers escape from quantum wells. From our experiments, comparing with open circuit, nearly 95% of the photo-carriers lost (considering the carriers for photoluminescence) when the p-n junction is under short circuit. Some documents have attributed this lost to the two main mechanisms of the carriers’ extraction from quantum wells, i.e. thermionic electron emission and tunneling mechanism^22^,^23^. However, the equivalent effect of these two mechanisms on carriers’ extraction should be measurable in multiple quantum wells with and without p-n junctions. This disagrees with our measurements when an external bias used to simulate the inner field of p-n junction is applied on multiple quantum wells without p-n junction. In our experiment, applying a 7 V external bias to the quantum well structure without a p-n junction only cause 22% lost of the photo-carriers. According to this, the carriers that are extracted from multiple quantum wells both with and without p-n junction by means of thermionic electron emission and tunneling mechanism are not more than 22 percent. So, the extra carriers’ lost can be attributed to the role of p-n junction, which means the carriers transportation was enhanced by p-n junction. This shows that applied an external bias to the quantum well structure without a p-n junction can’t implement the same effect of p-n junction. The p-n junction must have played some special role in carriers’ escaping from the quantum wells, thus the carrier absorption and transportation process should be discussed further. We will elaborate this phenomenon and mechanism in detail in another article. Here, the result of our experiments shows that the photo absorption and carriers transportation of InGaN/GaN quantum wells ware enhanced by p-n junction. Additionally, a reverse bias makes the carriers extraction much more distinct. This provides a new knowledge of absorption efficiency of quantum wells with p-n junction and demonstrates an innovative method of detector design.

In summary, we find that the photo excited carriers can escape from the quantum wells when the quantum wells are sandwiched in a p-n junction and the photon absorption of quantum wells is enhanced. This brings a better understanding of the physical characteristics of quantum well with p-n junction, and also gives us a new insight into the design of photodiode detector based on quantum wells.

## Methods

Epitaxial growth and chip fabrication. The samples used in this work are grown on c-plane sapphire substrates by a Veeco GaN MOVPE system. The trimethylgallium (TMGa), triethylgallium (TEGa), trimethylindium (TMIn) and ammonia (NH_3_) are used as precursors. Silane (SiH_4_) and biscyclopentadienyl magnesium (Cp_2_Mg) were used as n-type and p-type dopants, respectively. After the thermal treatment of sapphire wafers in H_2_ at 1050 °C, a 25-nm-thick GaN nucleation layer was deposited at 530 °C, followed by a 3.5-um-thick n-GaN(3 × 10^18^ cm^−3^) deposited at 1050 °C. Then 10 periods of InGaN/GaN (2.5 nm InGaN wells and 14 nm GaN barriers) MQWs were growth at 720 °C. The last layer for sample A (with p-n junction) is a 200-nm-thick p-GaN (5 × 10^17^ cm^−3^), and for sample B (without p-n junction) is a 200-nm-thick n-GaN (3 × 10^18^cm^−3^). After growth, the epi-wafers were fabricated to 1 mm × 1 mm area size chips for later measurements, using Ti/Cr/Ni and Ni/Au as the n-type contact and p-type contact, respectively.

Photo-electric measurement. A 405 nm semiconductor laser diode was selected as the resonant excitation source, the output power could be adjusted from 25 mW to 1 mW using a neutral optical attenuator. The PL spectra under open circuit condition and bias condition, open circuit voltage, photocurrent under bias condition are measured simultaneously. When sample A is under reverse bias condition, the p-type contact is connecting with the negative electrode of a constant voltage source and the n-type contact is connecting with the corresponding positive electrode. For sample B, the n-type contact above the quantum wells is connecting with the negative electrode and the n-type contact below the quantum wells is connecting with the positive electrode.

## Additional Information

**How to cite this article**: Yang, H. *et al*. The enhanced photo absorption and carrier transportation of InGaN/GaN Quantum Wells for photodiode detector applications. *Sci. Rep.* **7**, 43357; doi: 10.1038/srep43357 (2017).

**Publisher's note:** Springer Nature remains neutral with regard to jurisdictional claims in published maps and institutional affiliations.

## Figures and Tables

**Figure 1:**
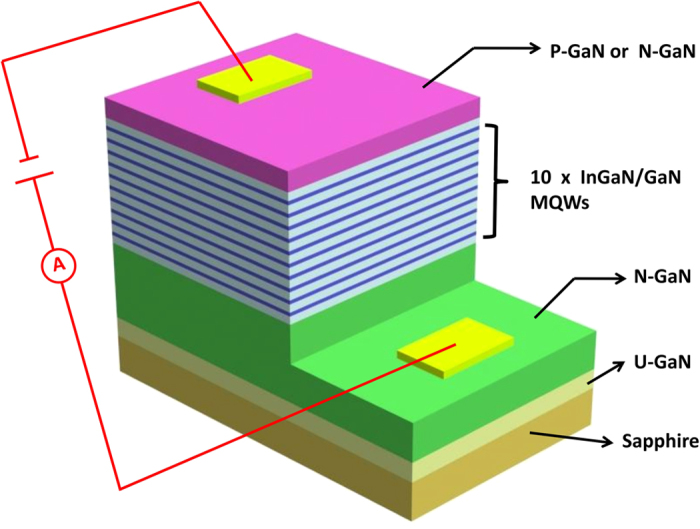
The schematic structure of samples studied in this paper. In sample A (with p-n junction), a p-GaN layer is deposited on top of the multiple quantum well structure while in sample B (without p-n junction), the top layer is n-GaN. To bias the sample, the top electrode is connected to the negative pole of a constant voltage source and the bottom n-type contact is connected to the corresponding positive electrode.
